# Augmented Reality Application for Handheld Devices: How to Make It hAPPen at the Pavia University History Museum

**DOI:** 10.1007/s11191-021-00197-z

**Published:** 2021-03-18

**Authors:** Lidia Falomo Bernarduzzi, Ester Maria Bernardi, Alberto Ferrari, Maria Carla Garbarino, Andrea Vai

**Affiliations:** 1 Physics Department and University History Museum, University of Pavia, Pavia, Italy; 2 Organization and Innovation Services, University of Pavia, Pavia, Italy; 3 Pavia University History Museum, University of Pavia, Pavia, Italy; 4 Physics Department, University of Pavia, Pavia, Italy

## Abstract

The Pavia University History Museum, which houses historic items mainly connected to the physics and medicine fields, has focused in the past years on new ways to involve its public and to attract new audiences. Among different approaches, digital technologies have proven important to both external and internal communication. Lately, an Augmented Reality application has been made available to visitors, offering in one tool multimedia material of a historical-scientific nature: stories, 3D animations, images and user-generated video storytelling (developed mainly by University students, one of our least present demographics before the App, and younger students, who typically participate in the annual co-creative project). The App was designed to be as non-intrusive and discreet as possible, to preserve the historic ambiance of the museum, to unite social and educational aspects, to register user behaviour and to make the museum experience more vibrant and active and therefore captivating.

## A Museum’s Needs

### What Does a Museum Want?

Offering visitors an outstanding and involving experience is one of the fundamental purposes of museums, and a task which may be difficult to accomplish in some cases. Museums without worldwide recognition, numerous staff and conspicuous budgets may find the task even more daunting. In order to make their communication effective and to strengthen their educational and social functions (encouraging participation and critical thinking and supporting inclusivity), museums have been turning to the latest technologies (Ferdani et al. 2015; Economou 2015; Economou and Meintani 2011; Ciolfi et al. 2011; Hammady et al. 2016; Bekele et al. 2018; Erb et al. 2012).

According to a poll conducted by the Osservatorio Innovazione Digitale nei Beni e Attività Culturali del Politecnico di Milano (Digital Innovation Observatory in Cultural Heritage and Activities of the Polytechnic of Milan) on the Italian museums, in 2019 “Virtual Reality (16%), Augmented Reality (12%) and videogames (10%) began spreading as a way to engage visitors and to interact with them. Seventeen percent of cultural institutions states that they offer an App and 62% expects to deploy one shortly” (Lorenzini 2019). But which technologies to select for any museums’ specific need, and how to implement them?

**Fig. 1:**
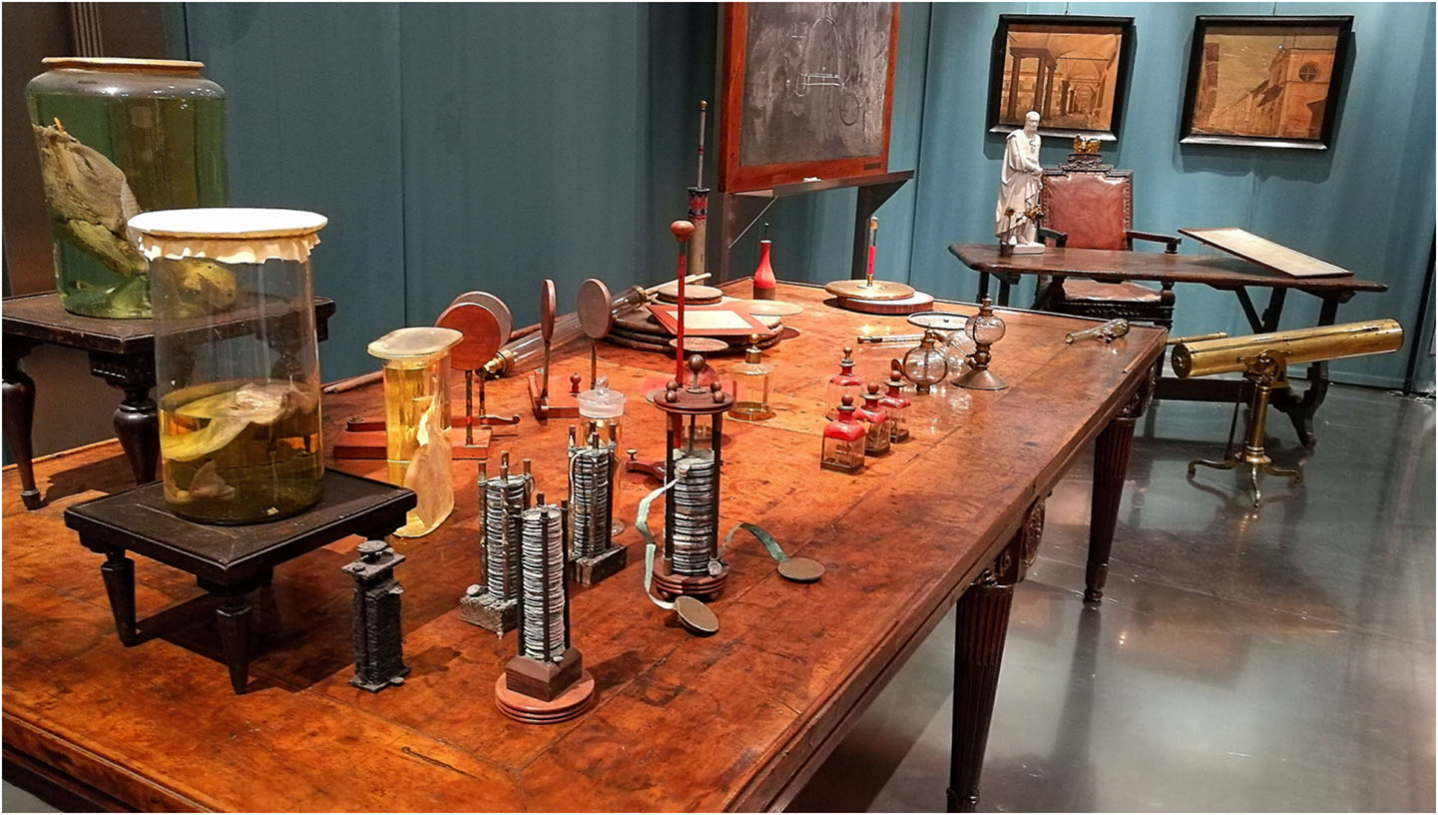
Alessandro Volta’s work table (with his inventions), his blackboard and desk

The Pavia University History Museum (Museo per la Storia dell’Università di Pavia, or MSU) faced this very same question in recent years. The specificity of the topics handled and of the items displayed made enhancing the exchange with the visitors and involving them a particularly difficult challenge. The MSU houses items connected to the history of the University of Pavia, mainly pertaining to the fields of Medicine and Physics (Fig. 1). On one hand, the large collection of anatomical preparations and medical instruments and the collection of Physics instruments designed or gathered by numerous scholars throughout the centuries constitute an invaluable asset; on the other, these have proven to be items in need of specific clarifications: historical reconstruction of their use, context of development and related scientific debates. Historical explanations, given with tact and sensitivity, can also help mitigate the unease that some visitors may feel when encountering human anatomical preparations, by highlighting their fundamental importance for the practice of medicine and surgery.

**Fig. 2:**
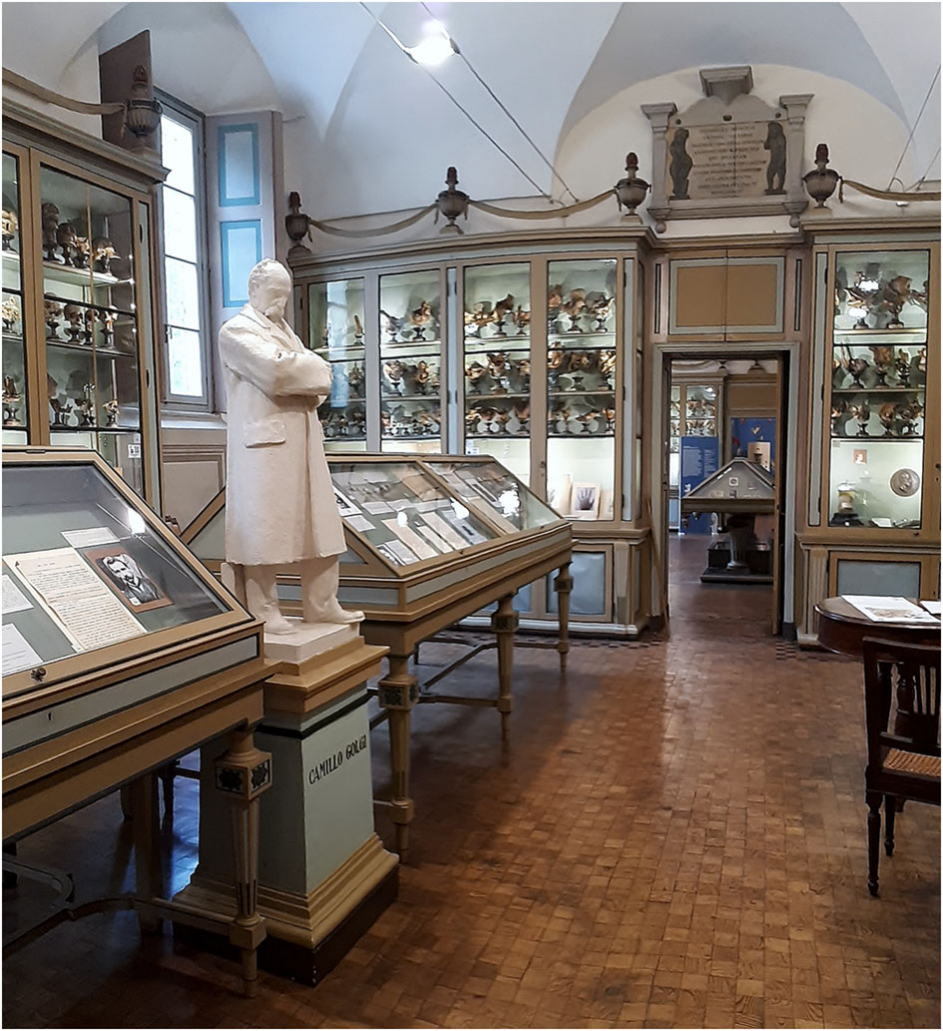
Golgi room

The MSU already offered audio and printed guides, as well as fact sheets, but it was not enough. The museum was still in need of a communication tool that allowed different types of public to access large quantities of material involving multiple types of media, to encourage the visitor to choose their preferred channel for in-depth information (Gardner 2011). Another element that needed to be considered carefully was the museum’s environment, also quite impressive (Fig. 2): the cabinets and shelves storing the collections are centuries old, and, in most cases, are also the original furnishings used to house the items. There was a concrete need to eliminate all signboards and written explanations, as they lessened the immersive feel that the museum aimed to inspire: ideally, the visitors should find themselves enveloped in a historic atmosphere, bringing forth a more authentic sense of the scientific activities of prominent scholars and inventors. Therefore, whichever technology was going to be selected as replacement for the physical signage needed to be as non-intrusive and discreet as possible.

Lastly, it was necessary to allow the public to customize the visit to their needs. Customization is key when it comes to large spaces and numerous exhibitions; the eye of the visitor can be caught by many visual stimuli and there needs to be an easy, quick and immediate way to address the sudden curiosity aroused in the viewer, lest they lose interest.

Instantly satisfying the curiosity of the viewer would also present the added benefit of taking into account another objective of museums: public participation. As the visitor’s need for information is efficiently and swiftly satisfied, a need for sharing and testifying to their experience is created; the visitors are stimulated to communicate their newfound knowledge to others and to enrich the content conveyed with their own observations, their own unique points of view. Allowing this need to find satisfaction creates value and enables participatory storytelling, which is vital to making museum experiences vibrant and active—and therefore captivating.

After all, as John Hartley and Potts (2014, p.70) note, “culture is the *survival vehicle* for groups [and] stories are the survival vehicle for culture”. Storytelling therefore plays a vital role in museums, as it is the perfect and most natural means of transmission for cultural heritage. Archaeologist Elisa Bonacini (2012, p. 98) delves deeply into the importance of making the visitor a participant in the communication effort to guarantee

[…] the definitive transformation of cultural institutions into “socio-cultural platforms of integrated development” […] which connect the actors involved (“creators, distributors, consumers, critics and collaborators”) and are open to cooperate with the users (even to the creation of museum materials) through personal user-generated content.

That is, in essence, the reason behind the continuous endeavour to involve visitors in the creation of stories in museums. All the more reason to pay special attention, in a university museum, to the visitors’ need to share experiences, for example through participatory storytelling: student visitors, particularly from the media, communication and literature fields, should be allowed to express their fresh takes on the contents and incited to explore current media formats and storytelling methods^1^. These visitors, indeed, possess specific skills and know-how that lend themselves beautifully to the creation of digital materials: other visitors may be guided by them in their museum exploration, and their experience may be enriched. So what kind of technology could accomplish all of this and still leave room for upgrading and improvement?

## The Contenders

### Technologies Up for the Role

Currently, there are quite a few technologies and tools that museums can adopt to reach their goals. Out of the many, the ones taken into consideration by the MSU were game application (game App), Quick Response codes (QR code), Virtual Reality application (VR App) and Augmented Reality application (AR App). A few considerations on these technologies, taken into account before making the final choice, are listed below.

#### Game App

Entertainment and education have long joined forces. From *edutainment*, however, the discussion in museum environments and in cultural heritage has moved on to the concept of *serious game* (Charsky 2010; Anderson et al. 2010; Mortara et al. 2014) and, more recently, to that of *gamification* (Ioannides et al. 2017).

The visitors’ intrinsic motivation to deepen their knowledge is increased by presenting a well thought-out and developed environment constructed around reward-based or, better yet, non-reward-based logic (Nicholson 2015). Gamification and serious games are therefore excellent options.

The museum had already made an attempt at creating a participative digital game, asking young visitors—students of an elementary class—to develop both the story and the actual structure^2^. With help from their teacher and from an industry expert (especially in code writing with the gaming software *Unity*), the students did generate a functioning final product, and learnt a lot in the process: they grew their logic and analysis skills and acquired a thorough knowledge of museum contents and of the history of physics (they were guided through experiments replicating those of Volta, Ampère and Faraday). They also derived strong motivation and a sense of accomplishment from having their finished product utilized by the general public: the game, aptly named “Clicking science”, is still downloadable for use on multiple platforms at https://maurovanetti.itch.io/lsiuc.

Nevertheless, the game produced was a small, simple one. Creating a complete game, with no glitches and complex structure and graphics, usually requires very skilled and specialized professionals and a considerable budget (often much too large for most small museums).

Furthermore, a game App usually encourages the user to follow a story (as simple and discreet as it may be) while acting according to a set of rules; however powerful a game App can be, then, it does not allow for the independent perusal of data at the user’s will, which in this case was in part what the MSU aimed to achieve. The MSU would therefore benefit from a game App only as an added tool, a second level of engagement of sorts; the first means of digital connection to the contents to be offered to the public should be something else.

#### QR codes

Quick Response codes are a widespread technology by some inscribed in the category of Augmented Reality; they are a type of barcode and act essentially as visual links to digital content: a website page, a text, an image^3^.

This technology, compared to the others here considered, is inexpensive. Also, QR code scanners are very light and common smart device applications, so there is no need for programming or software developing: it is highly probable that potential visitors would already have such an App on their device, or have at least already heard of QR technology—and therefore have no difficulty nor reluctance grasping the concept of it.

An important point against the use of QR codes, however, comes from their fragmentary nature and their reduced flexibility: once the data connected to the single QR code is downloaded, there is no interacting with it—unless the data stored in the code is a website address (whether it leads to a social network or any other form of interactive website), in which case the user would interact with the website directly: that would however detach the user from the items shown (Schultz 2013; Mogali et al. 2019; Wein 2014) and cause them to feel less connected to the environment. Not only that, but this technology also restricts the data stored in each code to about 3 kilobytes (Rouillard 2008) and could therefore not store certain typically heavier media such as videos. It also does not allow for navigating multiple content items or media types from one single code: either each different media content would have to be linked to its own code, even when referring to the same item or topic, or the code would have to link directly to a dedicated webpage with all the contents desired—not the most fluid of data browsing experiences.

Finally, their use would require placing a large amount of high contrast (and therefore very noticeable) squares in the MSU, deterring from the historic atmosphere preserved for the visitor.

#### VR App

A non-game application created with Virtual Reality technology would certainly gather enthusiastic reactions from the public; it is a tool that allows for endless variety of media output, provided that they are integrated in a cohesive manner with the virtual world created.

VR can be a complex concept to define, due to the many features that converge into it and to the psychological, social, cultural and spatial considerations that one can derive from it; it might do best to consider M. Carrozzino and Bergamasco’s (2010), p. 453) “technology-oriented” definition:

a complex technology which exploits more low-level technologies (such as computer science, 3D graphics, robotics, etc.) in order to create a digital environment which users feel completely immersed inside, and which they may interact with.

VR is indeed often associated with the use of equipment (head-mounted displays, visors, gloves…) that favours immersiveness and involves the user with all or some senses in the virtual world, successfully detaching their perceptions from the physical reality. This is both a point in favour and against: building a solely digital world invariably creates engagement and interest, but it also moves the visitor’s attention further away from the physical items displayed in front of them and it usually isolates the user and limits their interactions with other members of the group. The MSU especially wants to encourage the visitor to have a direct connection with the items, as in the eighteenth and nineteenth centuries the masterful attention to detail and beauty of construction of the instruments, anatomical preparations and wax figures was unrivalled. The emotional aspect lent by direct contact with the historical environment must be preserved, and interactions during a visit need to be encouraged.

#### AR App

This technology superimposes digital elements on a real-time view of the physical realm, augmenting the quantity of information available to the user, and makes use of equipment (digital camera, compass, GPS…) to identify visual or spatial cues to correctly overlay the most pertinent information. An application developed with AR technology allows the user to connect with both the physical reality and the additional information at the same time, making for a seamless and efficient museum experience.

This is an extremely versatile technology, as well—even more so than VR; it has been used with great success in many fields. An AR App can be designed to be used with immersive gear—usually just head-mounted smart-glasses—or with simple handheld devices—usually smartphones and tablets; that is a definite advantage, and in fact AR applications have become widely available on mobile devices (Yuen et al. 2011). Just like a game App and a non-game VR App, however, a non-game AR App can require longer production time and incur significant expenses.

Of course, as with VR, costs will vary enormously based on type of content (3D images and animations, for instance, would make the project more expensive) and whether they are already available or in need of being developed by the AR company. In the case of MSU, the 3D animations had already been created (more on that later…) and were only awaiting a suitable media carrier. As for videos, image galleries and texts, they were all going to be prepared and supplied by the MSU staff, so the AR developers would really only need to create the code of the App structure and the graphic user interface (UI).

A few words should be spent on the practical approach to making an AR App accessible to the public; the technology can be either marker-based or markerless. Though markers can be appropriately designed to better adapt to the environment, they are indeed a bit more intrusive than markerless alternatives; however, they allow better control of the link between item and content, at lower costs. Furthermore, AR markers support the use of 3D models.

## The Winner: Augmented Reality Technology With Markers

### Rationale

Augmented Reality technology layers context into the physical world with minimum intrusiveness. In fact, this feature makes it particularly suited to use in environments that need not be contaminated with additional physical elements. Using AR allows the furnishings and fixtures to stay untouched by wordy signs, obtrusive panels and repositionable stickers; in their stead, a small square marker, designed to look as non-invasive as possible, allows for easy access to all information pertaining to a selected item. Furthermore, the amount and type of information that can be associated to each AR marker is virtually limitless and allows for an interactive and varied experience, maintaining a constant connection to the items displayed in front of the visitor.

The MSU staff chose to develop an application for smartphone and tablet in light of the relative inexpensiveness and availability of such devices. Indeed, visors and other head-mounted gear may be more efficient in unfolding a seamless experience and mobile devices may very well be more distracting, but smartphones are so common that each visitor would have their own and be already familiar with its specifics; on the other hand, AR visors can be very costly and would only be available for a few visitors at a time. Furthermore, usage of applications on mobile devices is so widespread and frequent, that most if not all users would have no difficulty with the technology. One more point in favour of an AR marker App is that the content can be retrieved even after the visit simply by asking the museum staff for digital copies of the markers or by photographing them.

It was decided that the application must rely on a server to store all content, in order to keep the architecture slim and functional and the application itself small—and therefore more appealing for download. Once the application is downloaded, it can run smoothly and effortlessly by accessing the data on the University server through the free wireless connection provided. This meant however that multiple versions of the App would have to be developed, to succeed in working on different mobile operating systems; although, as International Data Corporation (IDC)—one of the premier global providers of market intelligence for information technology—notes, Android and iOs together currently constitute over 99% of the operating system market share: only two versions of the application would be necessary (Chau and Reith 2020).

## App Development Phases

The phases of the work coincided largely with those suggested by the Microsoft guide to the Mobile Software Development Lifecycle^4^: we will use this structure to schematize the description of our activities.

### Inception and Design

The general notion of implementing one of the technologies discussed above in the museum first became a concrete project around 2014. Between 1998 and 2004, the History of Physics and Physics Education Group (Department of Physics of the University of Pavia) had implemented digital videos of the most historically and educationally important instruments of the Volta Cabinet (Bevilacqua and Falomo 2009; Bevilacqua et al. 2002; Falomo Bernarduzzi and Cassani 2002); these videos were made available on CD Rom, or directly online through a virtual visit of the Physics Cabinet; in 2014, a member of the team (L.F.B.) conceived the idea of creating a tool to render the videos, as well as other content, easily available to the visitors.

The project was then extended to include the Medicine section, finally covering all 6000 items present in the museum. Each marker could in fact contain limitless information on an indefinite number of items: some of the markers, in the end, were connected up to 13 items. The virtually endless capacity of this technology represented a considerable asset for the museum.

At the outset, the team had to determine which kinds of content would be made available through the App. For Volta’s Physics Cabinet, of particular relevance because the room hosts a reconstruction of Alessandro Volta’s laboratory with original furnishings and instruments, a concise but eloquent text explaining the peculiarities of each item and an image gallery showing details of the instruments (also taken from historical texts and/or catalogues compiled by the instrument makers) would be essential; also, since visuals and user engagement have become such effective educational tools, the presence of videos (both museum and user-generated) would not go amiss. Videos produced by the museum would have to include

- three different series of 3D animations, one to accurately describe the instruments, one to show their functioning (using only sounds to bring the visitors and students to reflect and ask questions after the visit, in their classrooms as well) and another to explain the functioning and applications of the instruments;
- stories taken from the history of science (Klassen and Klassen 2014) such as debates, historical episodes of ethical, technological, political, institutional, gender relevance, as well as stories related to “science con-artists” (Allchin 2012).

Video storytelling produced by University students would then be added to the appropriate section. To the same section would be added stories told by students of all levels participating in the annual co-creative project (Falomo Bernarduzzi et al. 2014; Falomo Bernarduzzi et al. 2016; Falomo Bernarduzzi and Albanesi 2015).

**Fig. 3:**
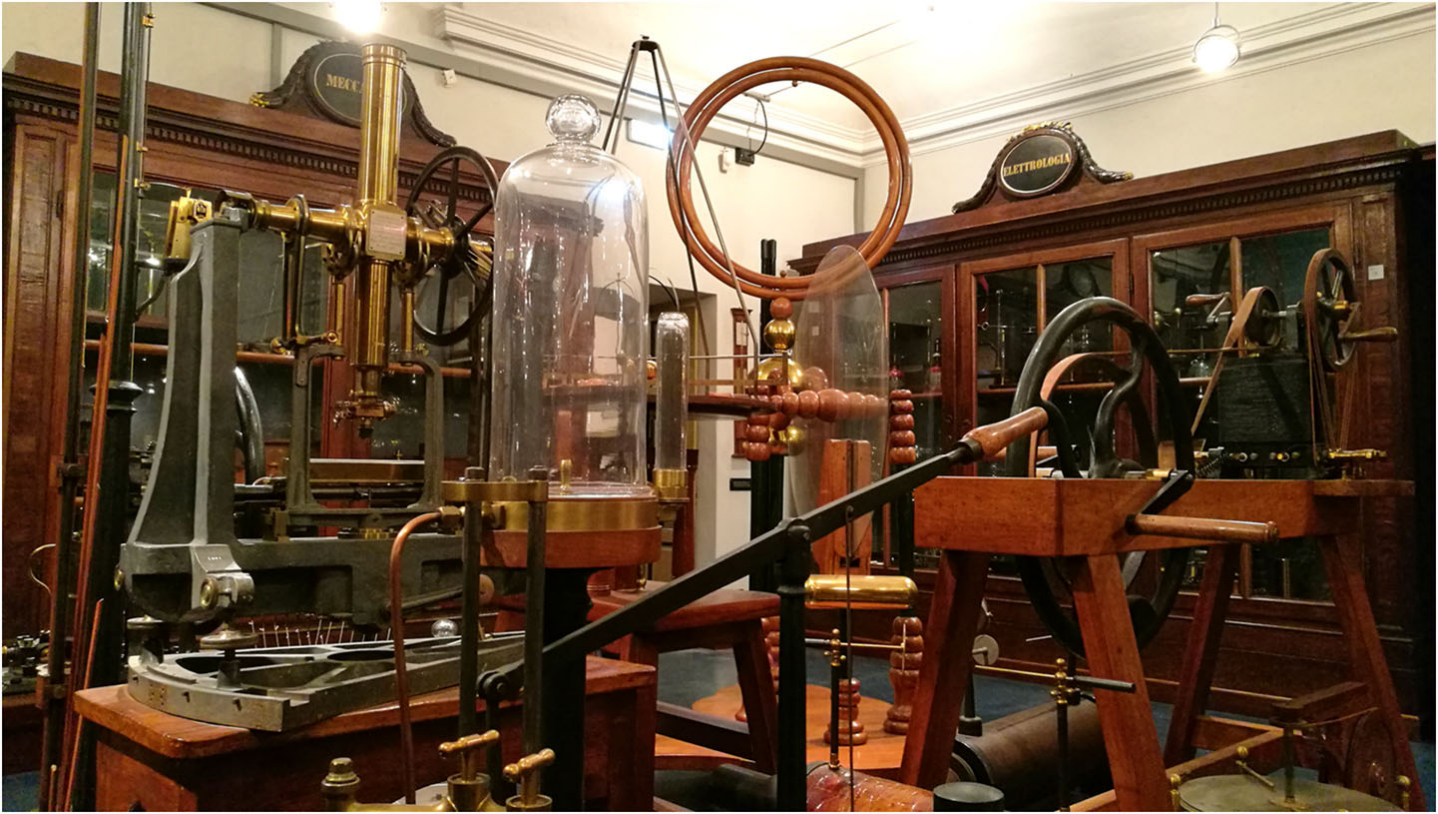
The 19th Century Physics Cabinet

The 19th Century Physics Cabinet (Fig. 3), the second room which composes the physics section of the museum and which houses a great number of instruments^5^, divided according to the different branches of physics (Mechanics, Acoustics, Pneumatics, Thermology, Thermodynamics, Optics, Electrostatics, Electrodynamics, Electromagnetism) in specific areas and shelves, required special consideration: the team opted to offer a general description of the historical evolution of each field during the course of the century and detailed descriptions of only a few items on display. Insights on historical figures from Pavia (who succeeded Volta to the Chair of Physics and as directors of the Physics Cabinet during the nineteenth century) were also made available.

Instead of 3D animations, AR markers in this Room link to Youtube videos produced by Paolo Brenni, the well-known physicist who restored the museum’s collections (Bellodi and Brenni 2001): Brenni put to work (https://www.youtube.com/user/florencefst) instruments of the Fondazione Scienza e Tecnica di Firenze (Brenni 2013), very similar to those on display in the 19th Century Physics Cabinet.

**Fig. 4:**
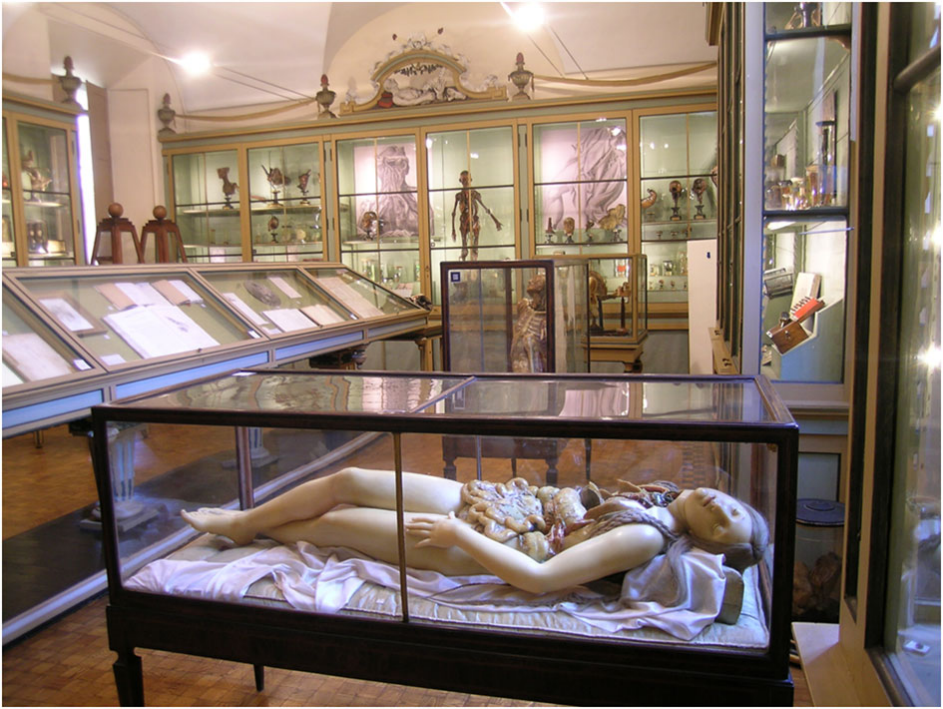
Scarpa room

As for the medical section, it was necessary to make content available to visitors in a different way than in the physics section. This depended, on the one hand, on the different structure of the narrative path of the rooms and, on the other, on the particular type of exhibited material, largely consisting of human anatomical preparations, which naturally require great forethought and sensitivity, also considering the strong emotional impact they could have on visitors. The rooms in the medicine section dedicate each display case to a more or less famous scientist (especially doctors and surgeons) active in the University of Pavia in particular historical moments. Each AR point, therefore, provides a short biographical profile of the scientist, combined with text that explain the choice of the pieces in the showcase (for example an important surgical intervention, the purpose for the making of an anatomical preparation or the events that led to the birth of a particular collection), historical images and short videos; in addition, there is the possibility of making illustrated volumes or important documents in relation to the exhibits virtually available, as well as showing the manipulation of objects that the visitor can only observe statically, because they are enclosed inside the showcases. An example is represented by the videos associated with some anatomical wax models, used between the end of the eighteenth century and the first half of the nineteenth century for teaching in the medical field (Fig. 4). The ceroplasmas were constructed in such a way as to be taken apart and observed in their individual parts, for better teaching effectiveness. Due to their fragility, however, they cannot be manipulated by visitors. Short videos would therefore be created and inserted into the App to allow visitors to observe the non-visible parts and how these models could be disassembled.

As for the functionality design, the team settled for a clean, simple aesthetic, with no launch screen, as the lightweight App was not expected to require much of a loading time on the landing screen. The landing screen was to be composed of the App icon and name in the upper left corner, the classic three-vertical-dot overflow menu icon on the upper right (leading to a Help screen) and the section selection options in the centre of the screen.

It was indeed decided that it would be more practical to have the App divided into content sections; the App managers could add as many sections as desired directly from the server at any given time and without need for extensive App structure re-programming. This ensured that, while at first only one trial section (Physics) would be implemented, more sections could be developed whenever required (the Medicine section, for instance, was inserted in December 2018). This furthermore gave way to the notion of extending the use of the App to other museums of the University of Pavia once the App had been launched, a notion which proved the flexibility and versatility of this arrangement. A future version of the App will most likely allow the use of GPS signals to automatically determine which museum the user is visiting.

By tapping on a section, the user would then make their selection and activate the loading of the available markers, visible as a colourful bar signalling percentage and numeric quantity of markers loaded. The visual cue was selected to account for different loading times on different devices: it was necessary to inform the user of the progress and make sure they understood that the App was neither frozen nor broken.

Once the markers were fully loaded, the camera of the device would be initiated; the user would point the device and frame a marker, and would then be prompted by a visual cue (i.e. the brightly lit contour of the marker) to tap on the marker. Upon doing so, icons representing the items and/or topics would appear—superimposed on real time environment by means of AR technology—allowing the user to make their selection by tapping on the screen.

After selecting the icon, the user would be given access to the content available for the specified item/topic. An exposed vertical menu would appear on the left side of the screen, displaying icons connected to actions: Back to previous, Text Section, Image Gallery, Museum Generated Videos and User Generated Videos. On the remainder of the screen, the Text Section would be displayed by default, easily changed by the user by tapping on any of the icons on the left menu.

On the whole, the team strived to create a design that would allow the user to seamlessly and effortlessly navigate between content, accessing them intuitively and independently. To aid in this effort, the brightly coloured icons were drawn to be clearly readable and of immediate understanding and all the elements were immersed in a high-contrast environment (i.e. white background paired with black text); whenever some content was not available, the team made sure that the icon reflected the fact, by appearing shaded in a dark grey hue. Now, how was the design, in effect, actualized?

## Development and Tuning

### How

App development began at the end of 2016/beginning of 2017. One of the main strong points of the entire project was utilizing the considerable resources available to the University by involving students; this tactic generates valuable products (which can be put to good use by the administration) while offering young learners the possibility to improve and put to the test the substantial skills provided them, in a relatively pressure-less environment. Part of the team were a Physics student and an Engineering student, who contributed to the development of an initial running code and who analysed the already existing optical recognition engine used to identify AR markers. After the first prototype was created, a specialized firm, MIDA Informatics, was involved. They converted the code project into a format which would then become the Android standard, and subsequently further developed the code, to integrate some specific aspects that were needed but still missing (multi-language and permanent camera activation during selections, for instance) and to turn the stub into a real App.

The firm also abstracted the data structure and prepared a content-delivering system on the server side, to allow the App to be more dynamic and easily extendible.

After completing the first Android prototype, MIDA proceeded with the development of the iOS version; using the same AR marker optical recognition technology already used for the Android one, they created an App with the same features, able to run on iOS.

The server content-delivering system was expanded to allow for transfer of localized data into any number of languages, but still preserving its completely dynamic structure. On both versions of the App, the interface was translated into English and Spanish, keeping the automatic selection of the interface language linked to the localization of the user’s device. At the same time, management of content in available languages was introduced in the App, keeping it a manual choice based on the actual availability of contents on the server.

A Google® Analytics® account was set up to monitor usage both for the Android and iOS versions (Ding 2017). The App is programmed to register user behaviour as a variety of “events”, which are then related to the Google Analytics Monitoring ID and made visible in the Events section of the system. The events that the App records are as follows: section selection, marker activation, item selection and activation of item detailed description as well as activation of image gallery, video gallery or user-generated video gallery and activation of video in player.

Lastly, the App was prepared for digital distribution services Google Play and Apple Store, by removing all the code not suitable for publishing and by disabling all of the more advanced debug systems (to ensure reliability and security on public stores). The App was named MuSt-UniPV (Museo Storico Università Pavia) and was then finally published on digital stores and made available to the public. The result of all operations was a Native application, expressed in Native code for both platforms (Android® Java® and iOS® Objective-C®)—with no use of cross-platform frameworks, web-Apps or any other hybrid technology.

A central role in the App is played by the marker recognition engine. It is a very powerful technology, as it allows for all the advantages previously discussed, but is also slightly fragile, as nearly all optical recognition systems are. The most troubling issues the team had to face were wrong outcomes caused by the inability of the marker recognition engine (and not depending on the App code) to recognize differences between some markers. Indeed, in some cases, the system would fail to distinguish one marker from another one, thus showing the user an unexpected/unintended instrument. Finding a solution to this issue was essential; after many attempts the team was able to identify and exclude the markers that posed the most problems and eliminate them from the project selection.

With the coding part completed, the team started focusing on inserting the content in the App database. The App accesses data on a dedicated University server, through a JavaScript Object Notation (JSON) file which describes the location of desired files and how to connect them to user actions. JSON syntax is relatively easy to understand, once the basics have been acquired, and is a format specifically developed for storing and transporting data (https://www.w3schools.com/js/js_json.asp).

A Communication student, who later worked as a Civil Service Volunteer and scholarship holder at the museum (A.F.), dedicated a consistent portion of his time to this assignment, eventually holding an important role in the project. He was tasked with learning the protocol and building up the database and JSON script, an assignment which required about 6 months to complete.

The App can be downloaded at

Android: https://play.google.com/store/apps/details?id=it.unipv.mustar&hl=en_US

Apple: https://apps.apple.com/ee/app/museo-storico-universit%C3%A0-pavia/id1383827031

**Fig. 5:**
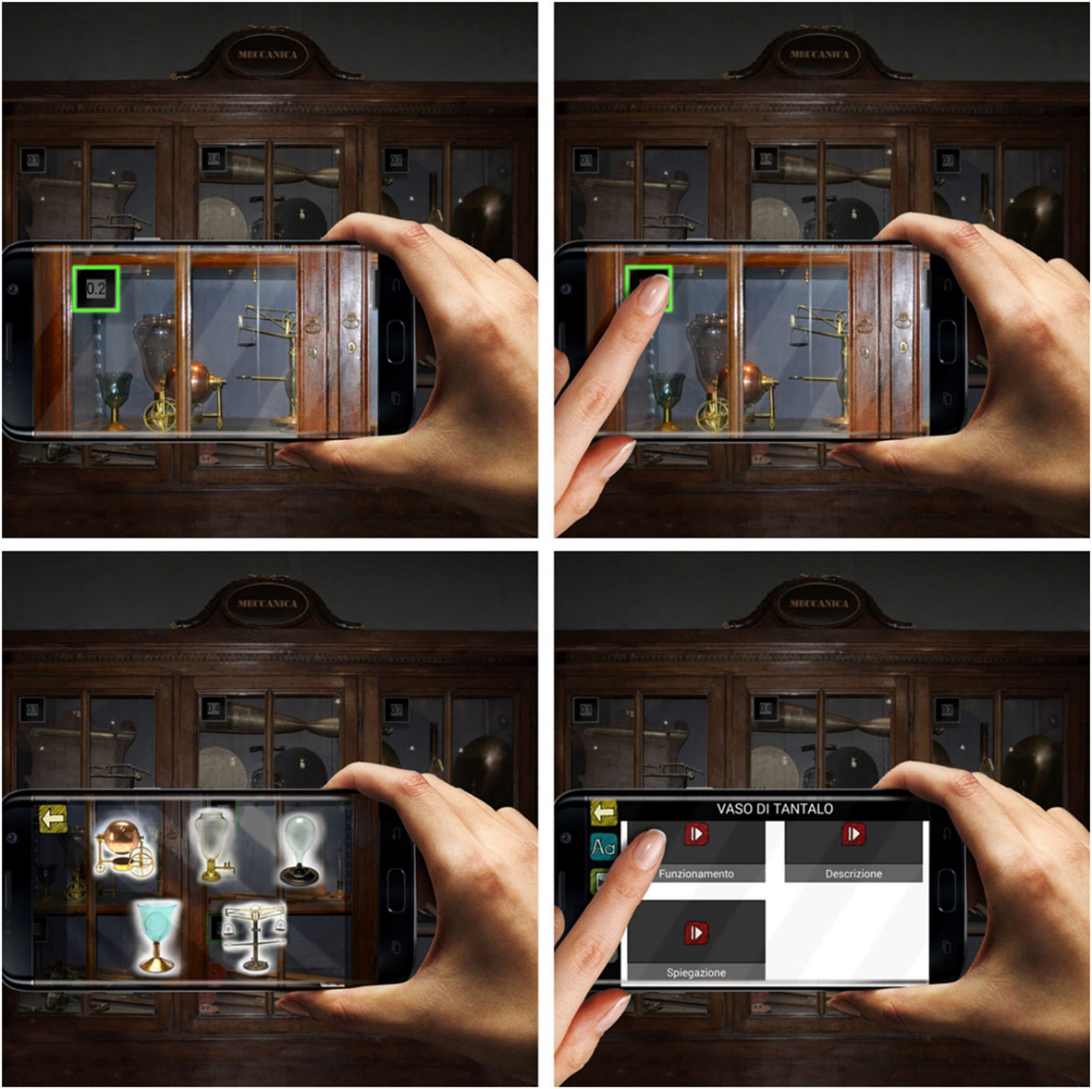
The figure shows the App in use; marker selection leads to the contents stored on the server

Once downloaded, the visitors will open the App and select the desired section, point the camera at the marker (Fig. 5) and tap on the screen to select. They can then browse at will. Some markers, at the moment, carry different information based on the museum section selected.

The process of inserting and updating content is an ongoing one; new media can be (and is) created and added at any time. Furthermore, while every item in the Volta cabinet is represented in the App, the remainder of the museum pieces had to undergo a sharper selection process, which means there is potentially a lot more media that could still be necessary to create and add to the App. In this respect, one must consider again the well of possibilities represented by a University context; Media, Literature and Foreign Languages students represent only a small portion of potential product creators that the MSU strives to engage in content development.

It might be worth mentioning that a few features that the team devised could not be implemented at the time of the App launch (such as a swipeable tutorial screen to give the visitors an onboarding experience); however, there are plans to include such functionalities in the very near future. Particularly interesting would be the introduction of fully interactive 3D models of the items; with this prospect in mind, the museum has already acquired (and begun experimenting with) a small 3D scanner.

## Deployment

To prepare for the big unveiling, the MSU staff proceeded to activate and test a free wireless internet connection. This operation proved quite challenging: the availability of the signal had to be restricted to museum grounds and Internet access could only be allowed to University servers and YouTube (where the user-generated content was uploaded). Furthermore, each device manufacturer had different security settings, which made for a more intricate procedure than anticipated: more than one technical strategy had to be employed to ensure optimal device activity. It also appeared there may be difficulties related to the network password (generated daily and posted on a board inside the museum): not all smartphones accepted the authentication process. To overcome this issue and to assist visitors who did not have a smartphone in the experience, the MSU staff made some tablets available, already prepared for use and tested for the App.

During this phase of experimenting, it appeared clear that the App had slightly different behaviours on different devices; for instance, when the camera was activated and engaging an AR marker, some devices showed a green rectangle on top of it, while others only contoured the marker in green and some did not give any indication that the marker was recognized by the camera at all. Still, internet connection aside, these were all minor differences, which did not pose any threat of disruption of the App experience.

## Conclusion

In the end, the long journey to the completion of the App was a highly informative and creatively stimulating one. The MSU team was enriched by the self-awareness resulting from the process of critically analysing the best approaches to implementing the new technology in the museum and was able to formulate new ways of introducing the collections to the visitors and to the students directly involved in the production of the storytelling videos.

The stories told by the museum guides constituted personal approaches to the topics, which were enhanced in the videos made by the students. Science stories have proven useful, as previously highlighted by other observers and experts, for learning about NOS content (historically contextualized) and human elements of science (Hansson et al. 2019), for communication of science to non-expert audiences and for engaging visitors (Dahlstrom 2014; Bedford 2001).

The University student visitors appreciated the opportunity to try their hand at making videos. Among the positive aspects reported by students of the Digital and Multimedia Communication course^6^ was a higher sense of belonging to a University of ancient origin and an understanding of the difficulties—not only of a technical nature—inherent in the diffusion of scientific culture. Indeed, some of them had a few difficulties in finding a compromise between the need to emotionally involve users and at the same time to provide information that adheres to historical “truth”, which requires a deepening of one’s understanding of the topic.

Analysis also proceeded from a quantitative standpoint; Google Analytics offers data as to the usage of the App. However, time periods should be studied keeping in mind a few observations.

From the launch of the application in December 2018 to the museum closing due to Covid-19 restrictions in February 2020, about 12 months of App usage taking into account normal scheduled closing times, analytics reports 456 App sessions, on a total of about 5000 visitors. It is worth noting, however, that in most cases the App is downloaded and used on one device which is then shared between two or more visitors; this further encourages discussion of museum topics and enhances the experience.

The low number of sessions may appear discouraging; there are, however, a few considerations to be made. Classes and groups, which constitute the majority of museum visitors, follow a guided tour. The type of communication favoured in these situations is definitely a more direct one between visitors and tour guide and few of the visitors have the opportunity of staying after the visit to further investigate topics that caught their attention.

Furthermore, in the last period before the closing of the museum due to the spread of Covid-19, it has been noticed that the App is used more frequently if the visit begins with replicas of historical experiments. The experience is highly involving and is based on the theme chosen for the visit, or on the interests of the class if the visit is a school field trip. Starting the visit with a highly involving activity, conducted with an inquiry learning approach—albeit with more “traditional” tools—leads to a greater degree of interest in multimedia contents presented later. As Heering (2017, p. 404) notes while describing experiments similar to those carried out at the museum: “Indeed, we have almost never encountered a group that was not ready to raise questions—people prove only too eager to receive contextualizing information about the experiments they have watched or participated in”. This statement rings particularly true, and could perhaps be extended to multimedia experiences; what needs to be further investigated is why visitors seem more likely to interact with the App when they are first involved in the experiments. In fact, when the experiments are conducted towards the middle of the visit (generally in the Physics room dedicated to Volta) the propensity to use the App is sensibly reduced. Perhaps a direct, more “tangible” involvement with historical tools and experiments, as well as a particular attention to dialogue as soon as the visitor enters the museum environment, would favour the establishment of a more relaxed atmosphere and dispel the hesitation in the use of software that is perceived as requiring commitment and maybe even failures.

Further, the more the visit coincides with the curricular topics dealt with at that time in the classroom, the more the students ask the staff to make the markers available for use outside the museum. It is worth noting that classes of students up to 16 years are in most cases not encouraged by the teacher to use the App (generally, in fact, for students up to 16 years of age, the use of smartphones is prohibited during lessons and outings). The App has shown its potential during the health emergency brought about by the Covid-19 pandemic.

During closure museum staff posted some markers on social media, allowing followers to use the application from their home without visiting the museum in person. The museum reopened in the middle of September; the historic period, with the spread of Covid-19 imposing a reduction of direct human contacts, has prompted staff to encourage visitors to use the application in lieu of fact sheets and audio guides: this of course allows users to enjoy a digitally guided tour of the rooms, free of charge and in full autonomy, by using their own device and while avoiding contact with surfaces and people. In the last period almost all visitors used the app.

Another parameter that offers interesting information is App usage duration. During the period between 15 December 2018 (launch of the App) and 30 October 2020, the App registered 680 sessions, with an average duration of 10 min and 22 s. At the two extremes of the range, Analytics reports that 71 sessions had an average duration of 50 min and 45 s, while 163 sessions lasted an average of less than 10 s. It is the opinion of the staff that this last data may signal a technical difficulty or an uncertainty that the user could feel towards activating the markers; this issue will be resolved in the coming upgrade of the App, with the addition of a few onboarding screens, a swipeable tutorial that will explain how to use the markers.

**Fig. 6:**
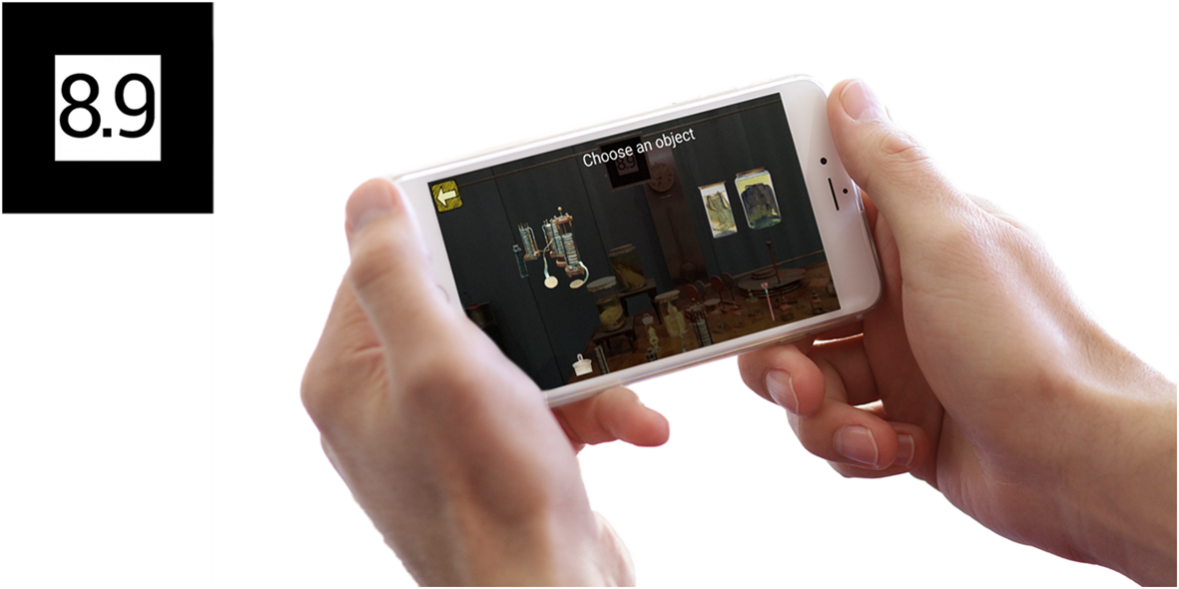
An example of marker from the Physics section. By framing the marker with the device camera and clicking on it, digital versions of Volta’s instruments will appear on the screen, superimposed to the view of the real instruments. The user can scroll down to view all the digital instruments available within the marker (as shown in the image to the right) and click on the instruments to view all pertaining content

**Fig. 7:**
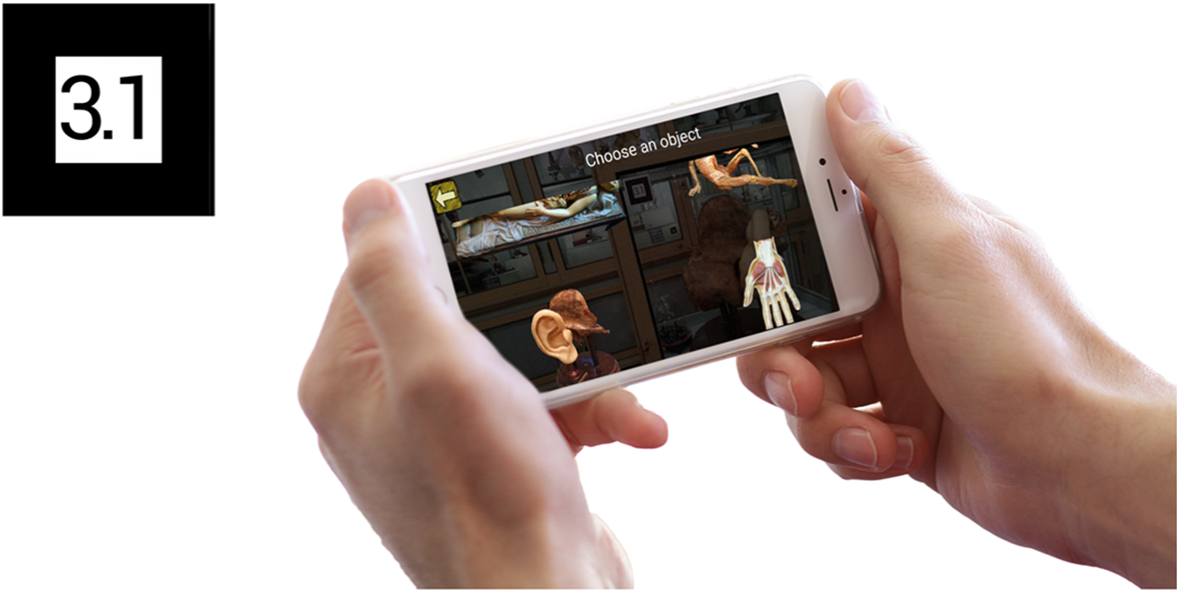
While in the medicine section, this marker will guide the user to the discovery of the ancient technique of ceroplastics

Foreign visitors seem to be more curious towards the museum App, and tend to use it for longer periods of time. Observing the data collected by Analytics, staff can also derive a sense of what captures the visitors’ attention; top events include, from the most occurring onwards, the explanation of the voltaic pile, of the X-rays, of the lantern microscope, of Volta’s pistol and of ceroplastics. The most selected AR markers are therefore in the physics section, with Alessandro Volta and his well-known invention, the battery pile, being the most viewed topics (Fig. 6). As for the medicine section, wax modelling (and in particular the female wax figure called “Venus”, showing the lymphatic system) appears to be most popular (Fig. 7).

Museum staff has started a revision process of the App, which will likely lead to some changes in the interface; a qualitative analysis has therefore been started, by asking visitors to write, on a voluntary basis, their feedback. From first data collected, we can infer that users found the App intuitive, effective and convenient; media such as historical images and videos were much appreciated. Following is a quote which reflects the general tone of most of the feedback sheets:

“*The App is useful to gain access to museum contents. It makes the visit more interactive and interesting. The addition of extra material only visible on the App is also important. In essence, it is an excellent idea to expand and better the material.*”

Visitors also noted a few issues that the staff will look into solving with the coming App upgrade: the download of markers which happens every time one needs to change museum section, slow loading of some videos. Furthermore, our effort to make the markers less invasive and to preserve the historical environment of the rooms was perceived by some younger visitors as a lack of visibility of the markers. Two Engineering students wrote

“*The App is a good idea, but improvable when considering the AR markers […] In essence, they are not very eye-catching*”.

There is still much that the MSU team aims to achieve; as noted, many features will be implemented to further enhance the reach and functionality of the App and more can be done to enlarge the pool of user-generated content. There is hope to extend the use of the App to the other museums in the Pavia University Museum System soon; the final goal would be having all six museums able to upload their own contents and to cooperate in the bettering of the App, in order to offer a customizable and attractive digital museum experience to all visitors.

## Footnotes

1 For instance, the director of the museum (LFB) is also a professor for the Digital and Multimedia Communication course at the University and she encourages students to create videos for the museum as part of the final examination.

2 Seymour Papert (1998, p. 88) stated, a good twenty years ago, how important it is “to encourage children to become game designers themselves. This requires more technological infrastructure and more support from knowledgeable people. But I have found that when they get the support and have access to suitable software systems, children’s enthusiasm for playing games easily gives rise to an enthusiasm for making them, and this in turn leads to more sophisticated thinking about all aspects of games *…*”

3 “A QR Code is a matrix code developed and released primarily to be a symbol that is easily interpreted by scanner equipment. […] Users photograph QR Codes and the software integrated into their phones decodes the messages and displays, manipulates, or stores the information on their mobile devices. […]” (Rouillard 2008, p. 2).

4 **Inception**—All Apps start with an idea. That idea is usually refined into a solid basis for an application; **Design**—The design phase consists of defining the App’s User Experience (UX) such as what the general layout is, how it works, etc., as well as turning that UX into a proper User Interface (UI) design, usually with the help of a graphic designer; **Development**—Usually the most resource intensive phase, this is the actual building of the application; **Stabilization**—When development is far enough along, Quality Assurance usually begins to test the application and bugs are fixed. Oftentimes an application will go into a limited beta phase in which a wider user audience is given a chance to use it and provide feedback and inform changes; **Deployment** (Ciclo di vita di sviluppo del software per dispositivi mobili 2016).

5 “… because of the rapid and spectacular progresses of physics and of the discovery of many new phenomena in the first half of the nineteenth century the number of available instruments increased very fast” (Brenni 2012 p. 199).

6 After presenting projects for examination, students were encouraged to express their opinion on the experience, both from a technical and personal point on view and with regards to the application of storytelling to scientific communication.
